# Intelligence

**Published:** 1829-02

**Authors:** 


### INTELLIGENCE.

### MONTHLY REPORT OF PREVALENT DISEASES.

During the course of the last month pulmonary affections have been frequent, but not marked by any great degree of severity. Many cases of rheumatism have fallen under our notice. We are still unable to detect the true intermittent form of this disease, which we have lately heard so loudly and so confidently laid down as its most usual type. In all the cases we have seen, calomel, opium, and colchicum, and moderate bleeding in the very early stages of the attack, have quickly cut short the disease. We have no confidence in any preparation of bark during the acute stages of rheumatism. When it has run on to a chronic state, the sulphate of quinine is frequently of much service.

Since our last report, peritoneal inflammation, of a low character, has occurred in several instances after easy and natural labours. These attacks have yielded to moderate bleeding *in the commencement*, and the free employment afterwards of calomel and opium, with occasional purgatives.

In three or four instances of slight paralytic affections, which have happened in old persons of broken-down constitution, much benefit has followed from the exhibition of ammonia and quinine, with, at the same time, local frictions and a mild yet nourishing diet.—Is not bleeding in such cases too indiscriminately had recourse to, from the prevailing belief that *every* modification of palsy, occurring either in the strong or the weak, indicates a disposition to apoplexy?

*Westminster Medical Society.* President, J. M. Arnott, Esq.—The following resolutions were passed unanimously at a special meeting of the society, held a short time ago.

Moved by Dr. J. Somerville. Resolved 1st. That it appears, from the

Report presented by the Committee on Anatomy to the House of Commons in the last session of Parliament, that, by the present state of the law, the only bodies which can be legally employed for dissection are those of persons executed for murder; and that even the possession of a body obtained in any other way is a misdemeanour.

Moved by Dr. WEBSTER. Resolved 2d. That this state of the law is injurious to students, teachers, and practitioners, in every department of medical and surgical science; and, in the opinion of the Committee of the House of Commons, is highly injurious to the public interests also.

Moved by Dr. MILLIGAN. Resolved 3d. That the measures recommended in the Report are, the repeal of any existing law which would subject to penalties those concerned in carrying the proposed plan into execution: the passing of an enactment permissive, but not mandatory, declaring that it shall not be illegal for the governors of workhouses, and other public institutions, to give up to dissection the bodies of those who die, without being claimed by their friends within a certain time: and the repeal of the clause of the Act of George II. which directs that the bodies of murderers shall be given up to be dissected.

Moved by J. R. BENNETT, Esq. Resolved 4th. That it appears to this Society that petitions should be presented to both Houses of Parliament, praying for some legislative measure which may give effect to the recommendations contained in the Report presented to the House of Commons in the last session.

Moved by G. JEWEL, Esq. Resolved 5th. That the committee be requested to draw up petitions founded on the preceding resolutions.

In compliance with the above resolutions, the following petition has been drawn up, and is now in course of signature by members. At an early day, it will be presented to the Commons by Mr. WARBURTON; that to the Lords, by the Marquis of LANSDOWN.

To the Honourable the Commons, &c.

The Petition of the undersigned Physicians, Surgeons, and Students of Medicine, Members of the Westminster Medical Society,

Humbly showeth, That, by the Report of the Select Committee of your Honourable House, appointed to inquire into the manner of obtaining subjects for dissection in the schools of anatomy, and the laws affecting the persons employed in obtaining and dissecting of bodies, bearing date the 22d day of July, 1828; the said Select Committee report, that the bodies of persons executed for murder are the only subjects which can legally be anatomised; and that the possession of a body for the purpose of dissection, obtained in any other manner, renders the possessor liable to an indictment for a misdemeanour; and the said Committee further report, that such a state of the law is, in their opinion, injurious to students, teachers, and practitioners, in every department of medical and surgical science, and highly prejudicial to the public interest.

Your petitioners therefore humbly pray your Honourable House, that the existing laws which direct the bodies of murderers to be anatomised, and which make the possession of a body for the purpose of dissection, obtained in any other manner, a misdemeanour, may be repealed. And that your Honourable House may be pleased to pass an enactment, enabling the governors of workhouses, and the directors of other public institutions, to dispose of, for the purpose of dissection, the bodies of those dying in workhouses or public institutions, such bodies not having been claimed within a limited time by any relative, or otherwise, as to your Honourable House may seem meet.

And your petitioners will ever pray.

---

*Machanic's Institution.*—On the 1st February, 1829, Dr. JOSEPH READE, author of "Experimental Outlines for a new Theory of Vision, Light, and Colours," will commence a course of lectures on Optics.

---

A patent has lately been granted to P. DERBISHIRE, of Ely place, Holborn, for a certain medicine or embrocation to prevent or alleviate sea-sickness, which may be usefully applied to other maladies.

We have heard of some cases in which this remedy is said to have been successful in preventing seasickness, in persons who had before always suffered very severely from it.—EDITORS.

---

Mr. GREEN, of Great Marlborough street, has lately published a very useful Chart of the Diseases of the Skin. The arrangement of the various diseases is in accordance with that of RAYER. The effects of the fumigating baths, as observed by Mr. Green during his very extensive experience, are stated in the last column.

---

Mr. BRANSBY COOPER, having been informed that Mr. LAMBERT asserted, at the last meeting of the Westminster Medical Society, that he had been employed to correct Mr. Cooper's work on the Ligaments, has requested us to state that he never employed Mr. Lambert for any such purpose; nor does he believe that that individual ever saw a line of the work until it was published.—*Medical Gazette*, Jan. 10th.

---

Mr. JAMES LAMBERT, reporter for the Lancet, has recently been expelled from the Westminster Medical Society, and also from the London Medical Society, by large majorities. Mr. Lambert is the person who wrote the libellous account of the operation for lithotomy which was performed by Mr. B. Cooper. A correct statement of this operation is given in the Hospital Reports of our present Number.

---

A very useful volume for the medical student has lately been published in Edinburgh, under the title of "The Medical Calendar, or Student's Guide." It contains a brief but very correct view of the various courses of medical and scientific instruction in the principal recognised schools of the United Kingdom and of Paris; and also an account of the rules to be observed for obtaining academical honours or professional diplomas, or for entering the public service. We would recommend this work to the attention of those who are commencing their professional education, either at home or in foreign countries.

---

Dr. HARINGTON has addressed a letter to a contemporary Journal, stating that he has for some time withdrawn himself from the Western Hospital, and that he has no further connexion whatever with that institution.

---

Mr. CÆSAR HAWKINS has been appointed assistant surgeon of St. George's Hospital.

### METEOROLOGICAL JOURNAL,

By Messrs. Harris and Co. Mathematical Instrument Makers, 50, High Holborn.

| December | Rain gauge. | Moon. | Thermom. 9 A.M. | MAX. | MIN. | Barometer. 9 A.M. | 10 P.M. | De Luc's Hygrom. 9 A.M. | 10 P.M. | Winds. 9 A.M. | 10 P.M. | Atmospheric Variations. 9 a.m. | 2 p.m. | 10 p.m. |
|---|---|---|---|---|---|---|---|---|---|---|---|---|---|---|
| 20 | | | 50 | 55 | 50 | 29.86 | 29.91 | 65 | 65 | NW | NW | Fine | Fine | Fine |
| 21 | | ○ | 53 | 55 | 50 | .93 | .93 | 65 | 64 | WNW | W | —— | —— | —— |
| 22 | | | 52 | 56 | 50 | .94 | .94 | 66 | 65 | N W | WNW | —— | —— | —— |
| 23 | | | 52 | 57 | 43 | .82 | .66 | 66 | 66 | N W | WNW | Sm. Ra. | Cloudy | Rain |
| 24 | | | 45 | 52 | 41 | .55 | .40 | 68 | 69 | WNW | W | Fo | Rain | —— |
| 25 | .16 | | 43 | 46 | 36 | .35 | .35 | 69 | 65 | WNW | WNW | —— | Cloudy | —— |
| 26 | | | 38 | 45 | 36 | .50 | .47 | 64 | 65 | WNW | W | Fine | —— | —— |
| 27 | | | 38 | 45 | 36 | .56 | .78 | 68 | 65 | SE | SE | —— | —— | Fine |
| 28 | | | 37 | 42 | 40 | .69 | .90 | 68 | 70 | SE | SW | Rain | Rain | Cloudy |
| 29 | | | 40 | 41 | 36 | 30.25 | 30.27 | 68 | 70 | SW | SW | Fog | Cloudy | Foggy |
| 30 | | ☾ | 37 | 42 | 39 | .26 | .15 | 69 | 67 | SW | SW | —— | —— | —— |
| 31 | .09 | | 40 | 48 | 38 | 29.97 | 29.86 | 66 | 68 | SSW | WSW | —— | Sleet | Rain |
| Jan. | | | | | | | | | | | | | | |
| 1 | | | 40 | 46 | 37 | .76 | .70 | 68 | 64 | W | NW | Fine | Cloudy | Fine |
| 2 | | | 40 | 42 | 36 | .78 | .85 | 62 | 65 | NW | NW | —— | Fine | —— |
| 3 | | | 38 | 42 | 38 | .80 | .84 | 63 | 64 | NW | NW | Sleet | Fine | Fine |
| 4 | | | 40 | 42 | 34 | .47 | .49 | 62 | 65 | NW | N | Fine | —— | —— |
| 5 | | ● | 35 | 38 | 29 | .60 | .75 | 64 | 67 | N | N | —— | —— | Snow |
| 6 | | | 30 | 35 | 29 | .88 | .88 | 67 | 69 | NNE | N | Snow | Fine | Sleet |
| 7 | | | 35 | 37 | 30 | .90 | .85 | 67 | 64 | NE | NE | Foggy | —— | Fine |
| 8 | | | 32 | 35 | 31 | .80 | .76 | 67 | 65 | NE | NNE | Cloudy | Cloudy | Snow |
| 9 | | | 33 | 35 | 33 | .69 | .55 | 67 | 66 | N | N | Foggy | Fine | Fine |
| 10 | | | 34 | 38 | 30 | .51 | .59 | 66 | 67 | NNE | NE | —— | —— | —— |
| 11 | | | 31 | 37 | 30 | .80 | .66 | 64 | 65 | E | E | —— | —— | —— |
| 12 | | ☽ | 36 | 38 | 32 | .87 | .88 | 67 | 66 | NE | NE | Foggy | Sleet | Cloudy |
| 13 | | | 34 | 28 | 33 | .97 | .98 | 65 | 63 | E | NE | Cloudy | Cloudy | Cloudy |
| 14 | | | 34 | 37 | 34 | .95 | .93 | 64 | 67 | NNE | NE | Fine | Fine | Cloudy |
| 15 | | | 35 | 38 | 31 | .82 | .65 | 67 | 66 | ENE | NE | Foggy | Cloudy | Cloudy |
| 16 | | | 32 | 34 | 27 | .52 | .54 | 65 | 66 | ENE | NE | Foggy | Fine | Fine |
| 17 | | | 29 | 30 | 25 | .65 | .77 | 67 | 64 | ENE | ENE | —— | —— | —— |
| 18 | | | 26 | 30 | 25 | .80 | .90 | 66 | 64 | ENE | E | —— | Foggy | Foggy |
| 19 | | | 26 | 30 | 25 | 30.01 | 30.01 | 65 | 65 | E | NE | Fog | —— | Fine |

The quantity of Rain fallen in the month of December, was 1 inch and 61.100ths.

